# Prevalence of cases of amebic liver abscess in a tertiary care centre in India: A study on risk factors, associated microflora and strain variation of *Entamoeba histolytica*

**DOI:** 10.1371/journal.pone.0214880

**Published:** 2019-04-03

**Authors:** Aradhana Singh, Tuhina Banerjee, Raju Kumar, Sunit Kumar Shukla

**Affiliations:** 1 Department of Microbiology, Institute of Medical Sciences, Banaras Hindu University, Varanasi, U.P., India; 2 Department of Gastroenterology, Institute of Medical Sciences, Banaras Hindu University, Varanasi, U.P., India; Centro de Investigacion y de Estudios Avanzados del Instituto Politecnico Nacional, MEXICO

## Abstract

**Background:**

Amebiasis, caused by *Entamoeba histolytica (E*. *histolytica)*, is a significant cause of morbidity and mortality in developing countries. Mortality due to amebiasis is mostly by extra intestinal infections, amebic liver abscess being the most common one. This study was conducted to determine the current epidemiological status, risk factors, associated microflora and strain variation of *E*. *histolytica* causing liver abscesses.

**Methods/Findings:**

A total of 115 liver abscess cases comprising of 107 (93%) males and 8 (6.9%) females were included in the study. Microscopic examination of pus samples from the abscesses and species discrimination using nested multiplex PCR showed the presence of *E*. *histolytica* in 101 (87.5%) cases. Data collected by face to face interviews using a pre tested questionnaire suggested intake of untreated drinking water (ORs: 6.4, p = 0.002), habit of alcohol consumption (ORs: 4.0, p = 0.019) and lack of urban services (ORs: 0.08, p = 0.017) to be major risk factors associated with *E*. *histolytica* infections. The study of associated bacterial flora through aerobic culture of liver aspirates and conventional PCR for detection of anaerobes revealed the presence of *Fusobacterium* (19, 25.5%), *Peptococcus* (19, 25.5%), *Prevotella* (18, 24.3%), *Bacteroides* (8, 10.8%), *Staphylococcus aureus* (3, 4%), *Escherichia coli* (2, 2.7%), *Peptostreptococcus* (2, 2.7%), *Clostridium* (2, 2.7%) and *Klebsiella pneumoniae* (1, 1.3%). Further to study the clonality, genotyping of *E*. *histolytica* targeting six tRNA-linked polymorphic STR loci (A-L, D-A, N-K, R-R, S^TGA^ -D and S-Q) was carried out which showed the presence of 89 different genotypes in the liver aspirate samples.

**Conclusion:**

The findings highlight the high prevalence of genetically diverse *E*. *histolytica* from the liver abscess cases in this geographical region. Low socio-economic status and habit of alcohol consumption were important predictors of amebic liver abscess.

## Introduction

Amebiasis is still a major health problem in tropical countries including India. Despite a decrease in mortality due to amebiasis by 14.8% over a decade [1] the number of affected persons has increased to 500 million people worldwide [2]. Mortality due to amebiasis is mostly by extra intestinal infections, amebic liver abscess being the most common one [3]. In majority of cases, this parasite remains as commensal in the intestine of humans but in some conditions it breaches the epithelial barrier and through blood supply reaches to different organs causing abscesses. Overall 10% of the world’s population is infected with *Entamoeba histolytica* (*E*. *histolytica*) but out of this only 1% becomes symptomatic. Nearly 20% of the Indian population show manifestations of the disease. [4].

It has been seen that majority of liver abscesses are polymicrobial in nature and *E*. *histolytica* is selective in its association with the bacterial population which in turn often aids in virulence [5]. As only a certain portion of individuals infected with *E*. *histolytica* develop liver abscess, it has been proposed that inter strain variations account for this behaviour of the parasite. Realising the limitations of microscopy and culture in detecting strain variations, PCR based genotyping is performed often targeting multiple loci to detect the allelic variations.

With this background, the present study was performed to determine the prevalence of *E*. *histolytica* causing liver abscesses and study the strain variations in and around Varanasi, North India. Additionally, the associated microflora of these abscesses was also assessed.

## Methods

### Ethics statement

The study was ethically approved by Institute Ethical Committee, Faculty of Medicine, Institute of Medical Sciences, Banaras Hindu University (EC Registration No. ECR/526/Inst/UP/2014 Dt. 31.1.14). The study included only adults and written informed consent was obtained from all the subjects who participated after explaining them the purpose of the study.

### Study population and sample collection

**Fig 1:**
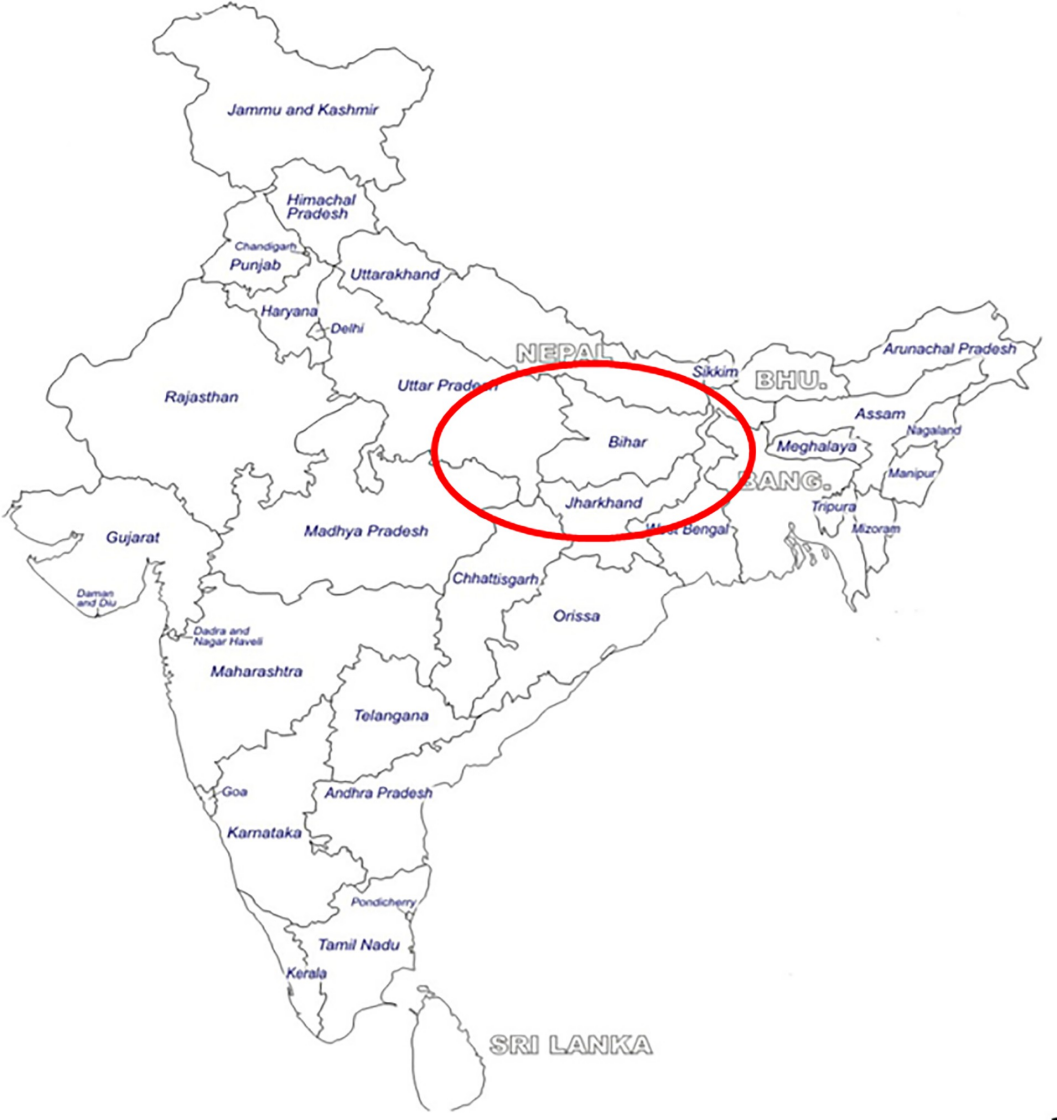
Geographical distribution of the population served by the tertiary care center.

This was a descriptive study conducted in the Department of Microbiology in collaboration with the Department of Gastroenterology of a tertiary care university hospital in Varanasi, India over a period of one year (January to December 2017). The tertiary care center is a premier 1500 bedded referral hospital catering to the medical needs of approximately 15 crores population from the states of Uttar Pradesh, Bihar, Jharkhand, Madhya Pradesh, Chattisgarh and neighboring countries Nepal and Bangladesh. The catchment area of the hospital has been depicted in Fig 1.

The study was initiated with collection of liver aspirates from 155 cases of liver abscess attending the Gastroenterology department. All these patients were subjected to ultrasound-guided drainage of the abscess due to medical indication. Further these cases were subjected to inclusion and exclusion criteria of the study. Well defined abscesses confirmed through abdominal ultrasonography, greater in size than 5 cm were included in the study. Patients with thick non-aspirable pus content, abscesses with size < 5 cm and multiple samples from the same patient were excluded from the study. Additionally, patients were instructed and requested to provide stool samples in dry sterile plastic containers. All the samples were brought to the laboratory immediately after collection and examined macroscopically and microscopically. Some portion of the sample was stored at -20°C for the DNA extraction. Relevant information was collected by face to face interviews using a pre tested questionnaire on demographic details (age, gender), socio-economic status and household characteristics of the study cases. The questionnaire used has been provided in the supplementary information (S1 Supporting Information). Laboratory data on alkaline phosphatase level (ALP), leucocytosis and status of anaemia were collected from the investigation records of the patients.

### Microscopy

Direct wet mount microscopy was performed for all the samples to screen for the presence of trophozoites only and trophozoites and cysts of *Entamoeba* in pus and stool samples respectively.

### Aerobic culture of liver aspirate

All the abscess aspirates were inoculated on Blood agar media and Mac Conkey agar and incubated at 37°C for overnight. Further growth on the culture plates was identified by colony morphology, Gram staining and standard biochemical tests [6].

### Genomic DNA extraction from the samples

The genomic DNA extraction was carried out using the modified CTAB-Chloroform method [7] and QIAmp DNA stool mini kit (Qiagen, Germany) as per manufacturer’s instructions from the liver aspirates and stool samples respectively. The extracted DNA was stored at -20°C for further processing.

### Nested multiplex PCR for detection of *Entamoeba* spp.

Detection of *Entamoeba* species in samples was done using nested multiplex PCR for *E*. *histolytica*, *E*. *dispar* and *E*. *moshkovskii* targeting the 16S-like rRNA gene [8]. PCR was performed in thermal cycler (BioRad, USA). Briefly 20 μL reaction mixture was used containing 2.5 μL of 10X reaction buffer (GeNei, Bangalore, India), 2.0 μL of 200 M concentrations of each of the deoxynucleoside triphosphates (dNTPs) (GeNei, Bangalore, India), 0.3 μL of 5U Taq DNA Polymerase (GeNei, Bangalore, India) and 1 μL of each oligonucleotide primers. Five microlitre (50 ng) of the DNA template and milli Q was added to maintain the final volume of 25 μL. In species specific nested round the amplicons from the genus specific primary round was used as template. The PCR mixture was subjected to an initial denaturation at 96°C for 2 minutes, followed by 30 cycles, each consisting of denaturation at 92°C for 60 seconds, annealing at 56°C for 60 seconds, extension at 72°C for 90 seconds and final extension at 72°C for 7 minutes. In the species specific round, the annealing temperature was set at 48°C. PCR products were examined for their expected base pair sizes on 1.5% agarose gel by loading 5 μl of the amplicons along with molecular marker of 100 bp ladder (GeNei, Bangalore, India).

### Conventional PCR for detection of anaerobic flora associated with liver abscesses

Six genera of anaerobes namely *Bacteroides*, *Peptococcus*, *Peptostreptococcus*, *Clostridium*, *Fusobacterium* and *Prevotella* that has been previously reported [9] were screened in the liver aspirates through conventional PCR using genus specific primers. The reaction mixture was as described earlier. The amplification conditions for *Bacteroides*, *Peptococcus*, *Peptostreptococcus* and *Clostridium* were; initial denaturation at 94°C for 5 min followed by 30 cycles of denaturation at 94°C for 30 sec, annealing temperatures 52°C, 53°C, 51°C and 60°C for *Bacteroides*, *Peptococcus*, *Peptostreptococcus* and *Clostridium* respectively for 1 min, extension at 72°C for 1 min, final extension at 72°C for 8 min [10]. For *Fusobacterium* the PCR conditions used was: initial denaturation at 94°C for 5 min, followed by 30 cycles of denaturation at 94°C for 30 sec, annealing at 60°C for 30 sec, extension at 72°C for 30 sec, followed by a final extension at 72°C for 7 min and for *Prevotella* the amplification program consisted initial denaturation at 94°C for 5 min; 40 cycles of denaturation at 94°C for 20 s, annealing at 55°C for 20 s, and extension at 72°C for 30 s; and final extension at 72°C for 5 min [11, 12]. PCR products were run on 1.5% agarose gel along with molecular marker of 100 bp ladder (GeNei, Bangalore, India).

### Species specific PCR to determine allelic variation and genotype assessment

PCR amplification was performed with *E*. *histolytica* specific primer pairs targeting six tRNA-linked polymorphic STR loci (A-L, D-A, N-K, R-R, S^TGA^ -D and S-Q) (Table 1) based on previous reference [13]. For the assessment of genotypes amplicons were designated a STR number based on their difference in sizes. The results from all the six STR loci were merged and a genotype number was assigned. The complete list of primers used in this study has been summarized in Table 1.

**Table 1:** Primer sequences used in the study.

S.No	Group	Name of primers	Primer sequence (5’-3’)
1.	Anaerobes	*Bacteroides*	F- GGGGTTCTGAGAGGAAG R- ACCCCCCATTGTACCAC
2.		*Peptostreptococcus*	F- AACTCCGGTGGTATCAGATG R-GGGGCTTCTGAGTCAGGTA
3.		*Peptococcus*	F- GGTGCCGCAGTAAACACAATAAGT R- AAGGCCCGGGAACGTATTCA
4.		*Clostridium*	F- CTCAACTTGGGTGCTGCATTT R- ATTGTAGTACGTGTGTAGCCC
5.		*Fusobacterium*	F- GAGAGAGCTTTGCGTCC R- TGGGCGCTGAGGTTCGAC
6.		*Prevotella*	F- CACRGTAAACGATGGATGCC R- GGTCGGGTTGCAGACC
7.	tRNA-linked polymorphic STR loci	AL3 AL5	GGATCGATACCCCTCATCTCCA CGCATCTTGCGATAGCCGAG
8.		D-A5 D-A3	CTGGTTAGTATCTTCGCCTGT GCTACACCCCCATTAACAAT
9.		N-K5 N-K3	CGAACGGCTGTTAACCGTTA TTCCTAGCTCAGTCGGTAGA
10.		R-R5 R-R3	AGCATCAGCCTTCTAAGCTG CTTCCGACTGAGCTAACAAG
11.		STGA-D5 STGA-D3	CTCTGGATGCGTAGGTTCAA GTATCTTCGCCTGTCACGTG
12.		S-Q5 S-Q3	GTGGTCTAAGGCGTGTGACT GAGATTCTGGTTCTTAGGACCC

### Statistical analysis

Statistical analysis was performed using 2018 Medcalc software (version: bvba) and SPSS version 19 (Armonk, NY). Odds Ratio (OR) was used to quantify the clinical profile of the cases and risk factors of *E*. *histolytica* associated liver abscess cases. Association of microflora with presence/absence of *E*. *histolytica* was compared by Chi-square test. Multiple correspondence analysis (MCA) was used to detect the pattern of association between a) the clinical parameters of the abscesses and bacterial diversity; b) the clinical parameters of the abscesses and genotypes of *E*. *histolytica*. The association pattern between the variables was inferred in terms of relative position of points along the dimensions. Points closer together were considered to be strongly associated. Values of p<0.05 were considered as significant.

## Results

### Prevalence of *Entamoeba* species

A total of 115 non repetitive cases of liver abscess from 107 (93%) males and 8 (6.9%) females were included and studied. Forty cases were excluded with reasons as mentioned: abscess size < 5 cm (n = 9), thick non-aspirable pus content (n = 11) and multiple samples from the same patient (n = 20).

**Fig 2:**
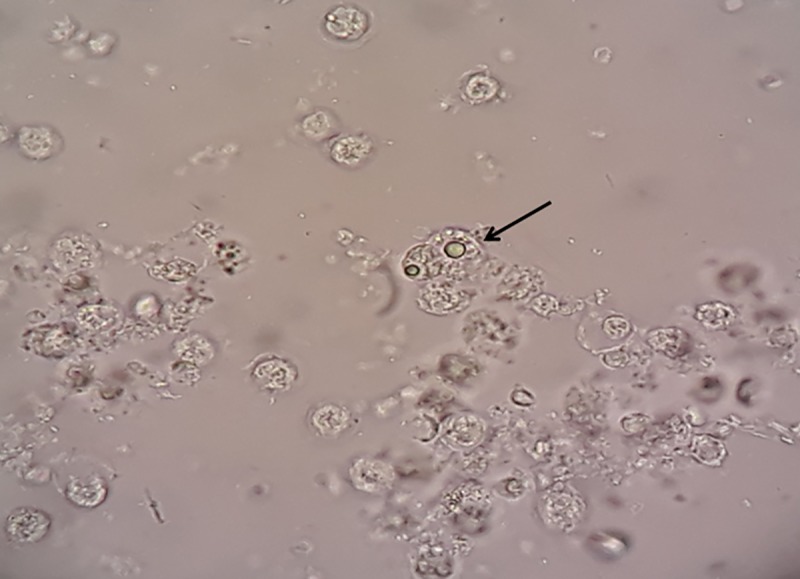
Microscopic image of trophozoite along with the pus cells.

**Fig 3:**
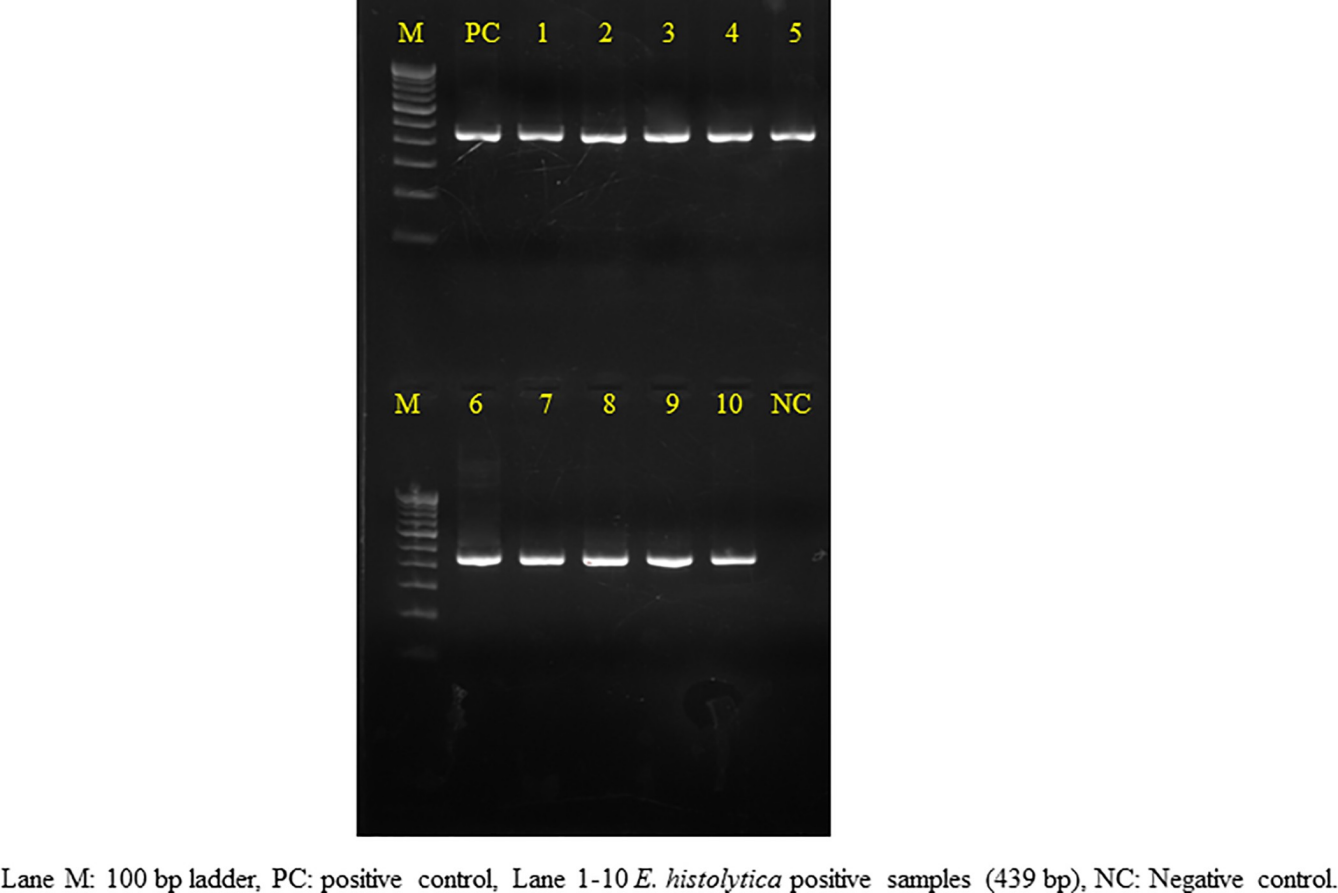
Agarose gel electrophoresis showing presence of *E*. *histolytica* in the liver aspirate samples.

Of these, only 6 (5.2%) liver aspirate samples were found to be positive for *E*. *histolytica / E*. *dispar* trophozoites through microscopy (Fig 2). However based on nested multiplex PCR, 87.8% (101/115) were found to be positive for *E*. *histolytica* (Fig 3). No other species of *Entamoeba* genus was found in any of the liver aspirates. Stool samples from all the patients could not be collected. Out of the 115 cases, 32 cases provided their stool samples for further analysis, of which none was found to be positive for *Entamoeba* cysts/ trophozoites through microscopy. However molecular detection showed the presence of *E*. *histolytica* in 4 (12.5%) samples and *E*. *dispar* in 1 (3.2%) sample. These four cases showing positive stool samples for *E*. *histolytica* also confirmed their presence in the respective liver aspirates.

When patient’s gender was considered, the prevalence of *E*. *histolytica* infections was found to be 93% (94) in males and 6.9% (7) in females and taking into account the socio-economic conditions the *E*. *histolytica* infected individuals were 62.3% (63) from low and 37.6% (38) from high socio-economic condition. A significant statistical difference in prevalence of the parasite was seen related with socioeconomic conditions (p = 0.003) but not with the gender (p>0.05) as shown in Table 2.

**Table 2:** Clinical profile and risk factors of *E*. *histolytica* associated liver abscess cases.

S.No.	Variables		No. (%)	Odds ratio (95% CI)	*P* value
1.	Age	< 41 years (n = 60)	54 (53.4)	1.5 (0.4–4.7)	0.458
		≥ 41 years(n = 55)	47 (46.5)		
2.	Gender	Male(n = 107)	94 (93.0)	1.0 (0.1–9.0)	0.976
		Female(n = 8)	7 (6.9)		
3.	Residence	Rural(n = 65)	52 (51.4)	0.08 (0.01–0.64)	0.017^≠^
		Urban(n = 50)	49 (48.5)		
4.	Education	No formal education(n = 45)	38 (37.6)	0.6 (0.1–2.5)	0.491
		Primary education(n = 30)	27 (26.7)		
		Higher education(n = 40)	36 (35.6)	0.6 (0.16–2.2)	0.449
5.	Drinking water	Untreated (n = 84)	79 (78.2)	6.4 (1.9–21.2)	0.002^≠^
		Filtered/Boiled (n = 31)	22 (21.7)		
6.	Habit of Alcoholism	YES(n = 75)	70 (69.3)	4.0 (1.2–13.1)	0.019^≠^
		NO(n = 40)	31 (30.6)		
7.	Lobe*	Right(n = 102)	93 (92)	7.3 (1.9–28.0)	0.003^≠^
		Left(n = 12)	7 (6.9)		
8.	Count of abscess	Single(n = 85)	78 (77.2)	3.3 (1.0–10.6)	0.036^≠^
		Multiple(n = 30)	23 (22.7)		
9.	Size of abscess	≤ average (n = 69)	61 (60.3)	1.1 (0.3–3.5)	0.816
		≥ average (n = 46)	40 (39.6)		
10.	Pus colour	Anchovy sauce (n = 70)	64 (64.6)	2.3 (0.7–7.1)	0.148
		Others (n = 45)	37 (37.3)		
11.	Level of ALP	Increased(n = 94)	87 (86.1)	6.2 (1.8–20.4)	0.002^≠^
		Normal(n = 21)	14 (13.8)		
12.	Condition of Leukocytosis	Present(n = 80)	72 (71.2)	1.8 (0.5–5.8)	0.286
		Absent(n = 35)	29 (28.7)		
13.	Anemia	Present(n = 67)	58 (57.4)	0.7 (0.2–2.3)	0.626
		Absent(n = 48)	43 (42.5)		

*Both lobes were affected in 1 case and was thus excluded from analysis.

≠ p<0.05

### Clinical characterstics and risk factors associated with *E*. *histolytica* infections

The overall prevalence of *E*. *histolytica* infections showed age dependency with significantly higher prevalence in the age group of 35 to 55 years (54.5%, 55). The mean age of participants was found to be 41.2 ± 14.3years. Abdominal pain in right upper quadrant, fever, vomiting, weight loss was seen in 80 (79.2%), 65 (64.3%), 9 (8.9%) and 10 (9.9%) cases respectively. However in a few cases diarrhea (8.9%, 9) and constipation (14.8%, 15) was also seen. Right lobe was the most common site of abscess formation as seen in 93 (92%) patients. There was only one case in which both lobes were involved. In 78 (77.2%) patients large, single abscess was seen, whereas in 23 (22.7%) cases there was presence of small multiple abscesses. The average size of the abscess was found to be 10.1 × 7.6 cm. In 14 (12.1%) cases the abscess was so enlarged that the patients were subjected to pig-tail drainage treatment instead of syringe aspiration. Increased level of alkaline phosphatase, leukocytosis and anemia was seen in 87 (86.1%), 72(71.2%) and 58 (57.4%) patients respectively. Though the exact occupation could not be revealed in majority of the cases, there was a dominance of unskilled group of workers (drivers, labors and factory workers).

The clinical profile and risk factors of *E*. *histolytica* associated liver abscess cases has been tabulated in Table 2. Intake of untreated drinking water (ORs: 6.4, p = 0.002), habit of alcohol consumption (ORs: 4.0, p = 0.019) and lack of urban services (ORs: 0.08, p = 0.017) were found to be major risk factors associated with the *E*. *histolytica* infections. Comparisons of the clinical profile of the cases with abscess parameters such as site of infection, count of abscesses, size of abscess, pus color and laboratory investigations revealed statistically significant association of the right lobe of liver (p = 0.003), single abscess (p = 0.036) and elevated levels of ALP (p = 0.002).

### Bacterial flora associated with liver aspirate

**Fig 4:**
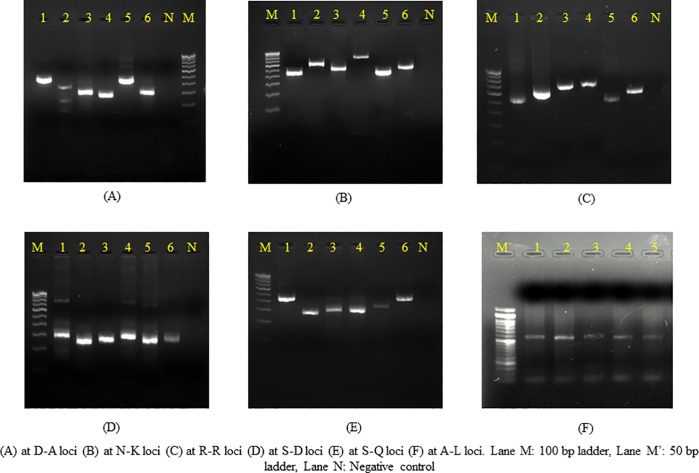
Agarose gel electrophoresis of different anaerobes in liver abscess samples.

**Fig 5:**
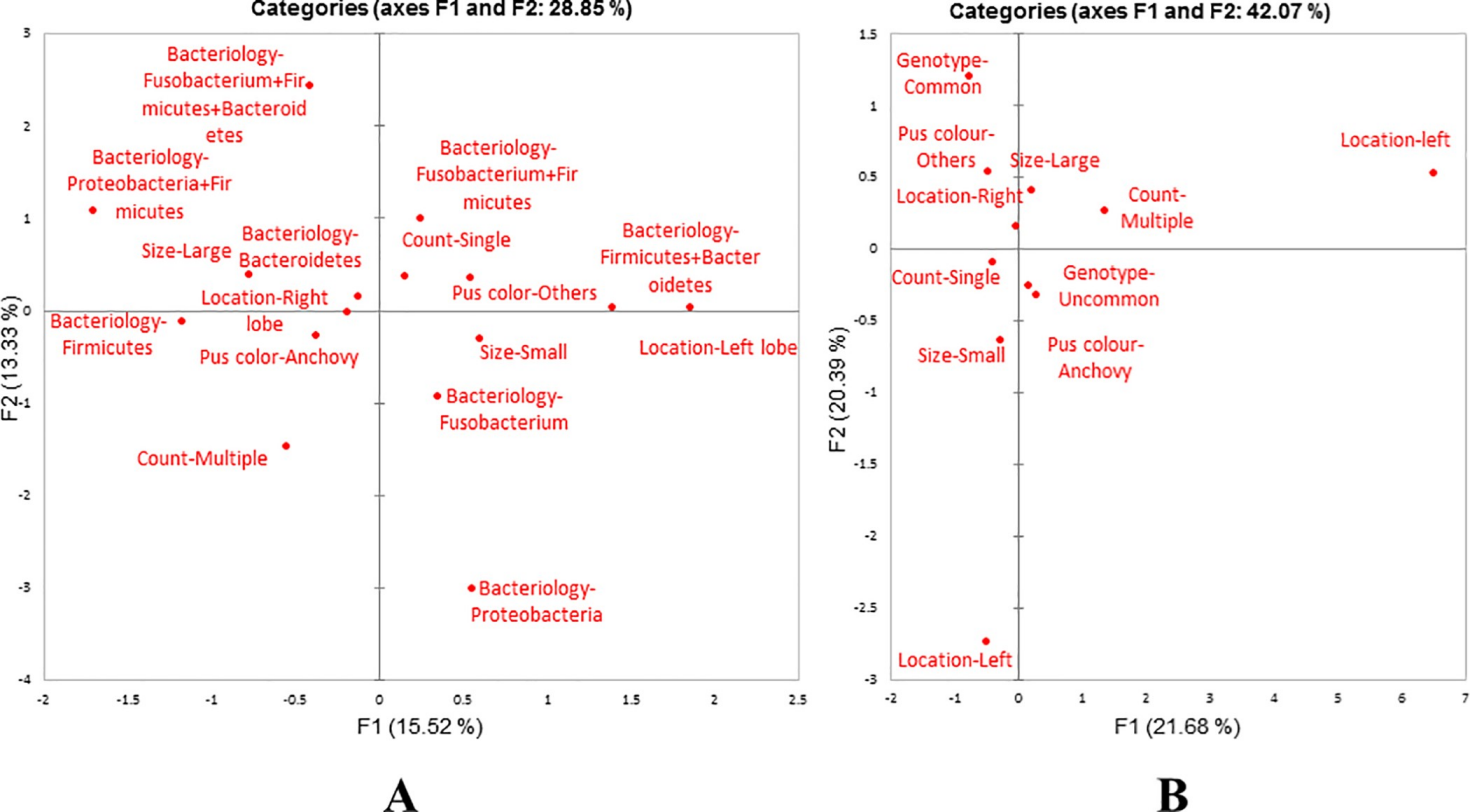
Multiple correspondence analyses of the association between (A) the clinical parameters of the abscesses and bacterial diversity; (B) the clinical parameters of the abscesses and *E*. *histolytica* genotypes.

Six (5.2%) samples showed the presence of bacteria namely *Staphylococcus aureus* (3, 50%), *Klebsiella pneumoniae* (1, 16.6%) and *Escherichia coli* (2, 33.3%) by culture. Among the 115 samples, 50 (43.4%) showed the presence of the different targeted anaerobes (Fig 4). The most common anaerobes was *Fusobacterium* (19, 27.9%) and *Peptococcus* (19, 27.9%), followed by *Prevotella* (18, 26.4%), *Bacteroides* (8, 11.7%), *Peptostreptococcus* (2, 2.9%) and *Clostridium* (2, 2.9%). In context of anaerobic bacterial flora, majority (74%) of the samples was monomicrobial, in which *Prevotella* and *Fusobacterium* was found most commonly in association with the *E*. *histolytica* as shown in Table 3. Ten samples that were negative for *E*. *histolytica* were also positive for anaerobic flora. The statistical comparison between the presence of microflora in *E*. *histolytica* positive and negative samples was not found to be significant (p > 0.05). Association between the clinical parameters of the abscesses and the bacterial phyla present, showed no correspondence by MCA (Fig 5A). The pattern of distribution described a limited association (28.85% for each dimension) among bacterial phyla and clinical parameters. No predictions for the presence of microflora could be made based on the clinical characteristics of the abscess.

**Table 3:** Association between *E*. *histolytica* and anaerobes present in the liver abscess fluid.

	Anaerobes^#^	Number (%)
*E*. *histolytica* Positive^#^	*Prevotella*	13 (19.1)
	*Fusobacterium*	12 (17.6)
	*Peptococcus*	3 (4.4)
	*Bacteroides*	2 (2.9)
	*Peptococcus + Bacteroides*	3 (4.4)
	*Peptococcus + Fusobacterium*	2 (2.9)
	*Peptococcus + Prevotella*	1(1.4)
	*Peptococcus + Prevotella + Peptostreptococcus*	2 (2.9)
	*Peptococcus + Fusobacterium + Bacteroides*	2 (2.9)
*E*. *histolytica* Negative^#^	*Peptococcus*	4 (5.8)
	*Prevotella*	2 (2.9)
	*Fusobacterium*	1 (1.4)
	*Peptococcus + Bacteroides*	1 (1.4)
	*Fusobacterium+ Clostridium*	1 (1.4)
	*Fusobacterium + Clostridium + Peptococcus*	1 (1.4)

^**#**^Results not significant, Chi-square test applied.

### Genotypes based on PCR amplification patterns at six t-RNA associated STR loci

**Fig 6:**
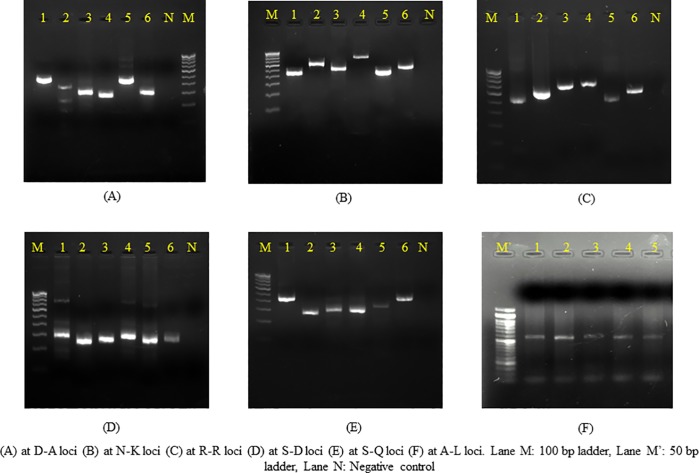
t-RNA linked STR pattern of selected *E*. *histolytica* samples.

Overall number of genotypes was very high. By combining the results from all the six markers, 89 genotypes were obtained in liver aspirate samples and four different genotypes in the stool samples of these patients. Seven genotypes (G2, G36, G39, G40, G47, G50 and G55) were found in more than one patient. Out of these genotypes G2, G36 and G50 were found in three patients and G39, G40 and G47 and G55 were found in two patients each. Maximum polymorphism was found in D-A and N-K loci. A single band was seen in majority of the samples however, in a few samples, second faint band was also observed (Fig 6). For simplicity, the band with more intensity was considered in the study. The genotypes of *E*. *histolytica* detected in the liver aspirate sample and stool sample of the same patients were different. Multiple correspondence analysis for the association (Fig 5B) between the clinical parameters of abscess and *E*. *histolytica* genotype showed that the common genotypes (found in more than one cases) were associated with right lobe of liver abscess with large abscess size and aspirate color different from the classical anchovy pus.

## Discussion

The present study showed a high overall prevalence rate of 87.8% of *E*. *histolytica* infections in the cases of liver abscess. This is in concordance with a similar study in our adjoining area which showed the prevalence of amebic DNA in 83.5% of the liver aspirate samples [14]. The sensitivity of PCR was higher as compared with microscopy in agreement with the other studies where PCR was found to be more reliable source of *E*. *histolytica* detection [14,15], the reason being that the *E*. *histolytica* is morphologically similar to *E*. *dispar* and *E*. *moshkovskii* and thus cannot be differentiated using microscopy. Additionally, *E*. *histolytica* trophozoites remain motile or viable for only a few minutes after coming in contact with air after aspiration, thus making the detection of this parasite by microscopy difficult. However, contrary to our results a study [16] showed microscopy to be more sensitive mode of detection of amebiasis. The reason behind the less sensitivity of PCR in the study was given to low parasite density and time related degeneration of trophozoites. In our study, high sensitivity of the molecular method can be attributed to use of nested PCR for the detection.

The occurrence of *E*. *histolytica* was predominant in the liver aspirates (87.8%) as compared to the stool samples (12.5%) of the liver abscess patients. Similar finding was presented in a study which demonstrated that stool examination and antigen detection test in stool samples are not useful in case of liver abscesses as most patients with this disease do not secrete detectable number of parasites in stool [17].

This study confirmed that the mid-aged males (mean age, 41.2 ± 14.3) are more susceptible to the liver abscess disease as previously reported by others [18] and the right liver lobe is the main site of infection as found in 92% of the cases. In concordance with our results, another study showed that such abscesses are 10 times more common in males as compared with females [19]. The reason for such sex discrimination can be attributed to the alcoholic hepatocellular damage which makes males more susceptible to the disease. Majority (77.2%) of liver abscess were solitary and large in size. Nevertheless, presence of multiple abscesses does not rule out the possibility of amebic liver abscess. Similar findings were suggested in a study where 577 adult cases of liver abscess were reviewed [20]. The classic description of an amebic liver abscess aspirated pus as thick paste called as ‘anchovy sauce’ has been overstressed in past, as in our study we found 64.6% aspirated pus as anchovy sauce while the remaining 37.3% was of other colors varying from brown to dirty yellow or ivory. Interestingly, all these pus colors were closely related to the presence of common genotypes of *E*. *histolytica* in these abscesses.

Previous studies had shown that factors like absence of urban services, inadequate hygienic practices and social determinants were associated with high prevalence of *E*. *histolytica* infections [21], Similarly, the present study confirmed that consumption of untreated contaminated water, lack of urban amenities and habit of alcohol consumption were significantly associated with cases of amoebic liver abscess.

Amebic liver aspirates have often been considered as bacteriologically sterile. However in most situations, they are mixed abscesses (originally caused by *E*. *histolytica* then infected by pyogenic bacteria) [22]. In our study 43 (37.3%) mixed abscesses were reported in which anaerobes such as *Fusobacterium* (27.9%), *Peptococcus* (27.9%) and *Prevotella* (26.4%) were found in abundance. However, no significant correlation between the bacterial microflora and the presence or absence of the parasite was found which might be due to the small number of *E*. *histolytica* negative samples in the study. Our results suggest that a high percentage of amebic liver abscess are coinfected with bacteria of intestinal microbiota which might have reached liver lobe together with the trophozoites from the large gut through portal circulatory system [22]. Studies have reported the non-specific mechanism of virulence modulation by bacteria in *Entamoeba*. In these reports, long term axenic culture of *E*. *histolytica* HM1:IMSS have shown the loss of virulence in *in-vitro* and *in-vivo* models of infection (amebic liver abscess in Hamsters) [23, 24]. The present study emphasizes the importance of screening for the predominant anaerobic microflora in case of liver abscesses. On the basis of multiple correspondence analysis, clinical parameters of the liver abscess did not relate with the type of bacteria which could be due to abundance of bacterial flora associated with the liver abscesses.

There are many strain identification tools available for the study of the genomic variability of *E*. *histolytica*, but each has its own limitations. Isoenzyme analysis can detect only a limited diversity and it also needs viable culture of the parasite. Other DNA based typing methods are available using serine rich Entamoeba histolytica protein (SREHP), chitinase gene but they either require use of restriction enzymes or sequencing of amplicons to detect genomic variation.

Using genotyping based on multi loci, we were able to demonstrate that *E*. *histolytica* has an extremely complex polymorphic genetic structure. A high degree of polymorphism was observed, despite the fact that the samples were from a restricted geographical location. By means of six t-RNA linked STR loci, a total of 89 genotypes were seen in the liver abscess samples. This level of diversity has been seen in previous reports where 85 genotypes have been detected in 111 unrelated samples [25]. A study from Bangladesh reported 25 different genotypes in 42 intestinal isolates and 9 genotypes among 12 liver abscess isolates using nested PCR of SREHP gene coupled with restriction digestion [26]. In another study based on sequencing of four different loci (chitinase, SREHP and two t-RNA linked loci) a total of 53 genotypes were seen in 63 samples [27]. Such high levels of genomic diversity suggest that there is rapid generation of new variants in the case of *E*. *histolytica* [25]. Thus, there must be some novel mechanism responsible for such a higher genomic variability. It was interesting to note that *E*. *histolytica* from liver aspirates showed different genotypes when compared with the intestinal samples in the same patient. This finding however, was limited to only in few cases due to non-availability of stool samples from all the patients of Amebic liver abscess, which is one of the major limitations of this study. Nevertheless, this difference in the genome of the *E*. *histolytica* strains between the intestinal and non-intestinal samples, hints towards its role in the partial virulence of this parasite. However, this can be better predicted when DNA based typing method will be used in different clinical outcomes of the *E*. *histolytica* infection cases, symptomatic and asymptomatic, to confirm whether genome plays any role in the differential virulence of this parasite.

## Supporting information

S1 STROBE Checklist (DOC)

S1 Supporting Information (PDF)

## References

[1] Wang H, Naghavi M, Allen C, Barber RM, Bhutta ZA, Carter A, et al Global, regional, and national life expectancy, all-cause mortality, and cause-specific mortality for 249 causes of death, 1980–2015: a systematic analysis for the Global Burden of Disease Study 2015. Lancet. 2016;388(10053):1459–544. 10.1016/S0140-6736(16)31012-1 27733281 PMC5388903

[2] Das SK, Chisti MJ, Malek MA, Salam MA, Ahmed T, Faruque AS, et al Comparison of clinical and laboratory characteristics of intestinal amebiasis with shigellosis among patients visiting a large urban diarrheal disease hospital in Bangladesh. Am J Trop Med Hyg. 2013;89(2):339–44. 10.4269/ajtmh.12-0570 23775017 PMC3741257

[3] Haque R, Huston CD, Hughes M, Erik H, Petri WA Jr. Amebiaisis. N Engl J Med. 2003;348:1565–73. 10.1056/NEJMra022710 12700377

[4] Rani R, Murthy RS, Bhattacharya S, Ahuja V, Rizvi MA, Paul J. Changes in bacterial profile during amebiasis: demonstration of anaerobic bacteria in ALA pus samples. Am J Trop Med Hyg. 2006;75(5):880–5. 17123981

[5] Mirelman DA. Ameba-bacterium relationship in amebiasis. Microbiol Rev. 1987;51(2):272 2885733 10.1128/mr.51.2.272-284.1987 PMC373106

[6] Winn WC. Koneman's color atlas and textbook of diagnostic microbiology 6^th^ ed Lippincott williams&wilkins; 2006.

[7] Parija SC, Khairnar K. Detection of excretory Entamoeba histolytica DNA in the urine, and detection of E. histolytica DNA and lectin antigen in the liver abscess pus for the diagnosis of amoebic liver abscess. BMC Microbiol. 2007;7(1):41. 17511859 10.1186/1471-2180-7-41 PMC1885440

[8] Khairnar K, Parija SC. A novel nested multiplex polymerase chain reaction (PCR) assay for differential detection of Entamoeba histolytica, E. moshkovskii and E. dispar DNA in stool samples. BMC Microbiol. 2007;7(1):47. 17524135 10.1186/1471-2180-7-47 PMC1888694

[9] Brook I, Frazier EH. Microbiology of liver and spleen abscesses. 1998;47(12):1075–80. 10.1099/00222615-47-12-1075 9856643

[10] Rekha R, Alam Rizvi M, Jaishree P. Designing and validation of genus-specific primers for human gut flora study. Electron J Biotechnol. 2006;9(5):0

[11] Nagano Y, Watabe M, Porter KG, Coulter WA, Millar BC, Elborn JS, et al Development of a genus-specific PCR assay for the molecular detection, confirmation and identification of Fusobacterium spp. Br J Biomed Sci. 2007;64(2):74–7. 17633142 10.1080/09674845.2007.11732760

[12] Matsuki T, Watanabe K, Fujimoto J, Miyamoto Y, Takada T, Matsumoto K, et al Development of 16S rRNA-gene-targeted group-specific primers for the detection and identification of predominant bacteria in human feces. Appl Environ Microbiol. 2002;68(11):5445–51. 10.1128/AEM.68.11.5445-5451.2002 12406736 PMC129894

[13] Ali IK, Zaki M, Clark CG. Use of PCR amplification of tRNA gene-linked short tandem repeats for genotyping Entamoeba histolytica. J Clin Microbiol. 2005;43(12):5842–7. 10.1128/JCM.43.12.5842-5847.2005 16333065 PMC1317169

[14] Jaiswal V, Ghoshal U, Baijal SS, Mittal B, Dhole TN, Ghoshal UC. Evaluation of antigen detection and polymerase chain reaction for diagnosis of amoebic liver abscess in patients on anti-amoebic treatment. BMC Res Notes. 2012;5(1):416. 22870930 10.1186/1756-0500-5-416 PMC3477060

[15] Mirelman D, Nuchamowitz Y, Stolarsky T. Comparison of use of enzyme-linked immunosorbent assay-based kits and PCR amplification of rRNA genes for simultaneous detection of Entamoeba histolytica and E. dispar. J Clin Microbiol. 1997;35(9):2405–7. 9276425 10.1128/jcm.35.9.2405-2407.1997 PMC229977

[16] Sri-Hidajati BS, Basuki S, Pusarawati S, Kusmartisnawati K, Rossyanti L, Sulistyowati SW, et al Comparison of multiplex single round PCR and microscopy in diagnosis of amoebiasis. Afr J Infect Dis. 2018;12(1S):120–6. 29619442 10.2101/Ajid.12v1S.18 PMC5876774

[17] Fotedar R, Stark D, Beebe N, Marriott D, Ellis J, Harkness J. Laboratory diagnostic techniques for Entamoeba species. Clin Microbiol Rev. 2007;20(3):511–32. 10.1128/CMR.00004-07 17630338 PMC1932757

[18] Farhana F, Jamaiah I, Rohela M, Abdul-Aziz NM, Nissapatorn V. A ten year (1999–2008) retrospective study of amoebiasis in University Malaya Medical Centre (UMMC), Kuala Lumpur, Malaysia. Trop Biomed. 2009;26:262–6. 20237439

[19] Shirley DA, Farr L, Watanabe K, Moonah S. A Review of the Global Burden, New Diagnostics, and Current Therapeutics for Amebiasis InOpen forum infectious diseases 2018(Vol. 5, No. 7, p. ofy161). US: Oxford University Press 10.1021/acsinfecdis.8b00298 PMC6055529 30046644

[20] Lodhi S, Sarwari AR, Muzammil M, Salam A, Smego RA. Features distinguishing amoebic from pyogenic liver abscess: a review of 577 adult cases. Trop Med Int Health. 2004;9(6):718–23. 10.1111/j.1365-3156.2004.01246.x 15189463

[21] Benetton ML, Gonçalves AV, Meneghini ME, Silva EF, Carneiro M. Risk factors for infection by the Entamoeba histolytica/E. dispar complex: an epidemiological study conducted in outpatient clinics in the city of Manaus, Amazon Region, Brazil. Trans R Soc Trop Med Hyg. 2005;99(7):532–40. 10.1016/j.trstmh.2004.11.015 15869773

[22] Reyna-Fabian ME, Zermeno V, Ximenez C, Flores J, Romero MF, Diaz D, et al Analysis of the bacterial diversity in liver abscess: differences between pyogenic and amebic abscesses. Am J Trop Med Hyg. 2016;94(1):147–55. 10.4269/ajtmh.15-0458 26572872 PMC4710420

[23] Wittner M, Rosenbaum RM. Role of bacteria in modifying virulence of Entamoeba histolytica. Am J Trop Med Hyg. 1970;19(5):755–61. 4318244 10.4269/ajtmh.1970.19.755

[24] Pimenta PF, Diamond LS, Mirelman D. Entamoeba histolytica Schaudinn, 1903 and Entamoeba dispar Brumpt, 1925: Differences in their Cell Surfaces and in the Bacteria‐containing Vacuoles. J Eukaryot Microbiol. 2002;49(3):209–19. 12120986 10.1111/j.1550-7408.2002.tb00525.x

[25] Ali IK, Mondal U, Roy S, Haque R, Petri WA, Clark CG. Evidence for a link between parasite genotype and outcome of infection with Entamoeba histolytica. J Clin Microbiol. 2007;45(2):285–9. 10.1128/JCM.01335-06 17122021 PMC1829016

[26] Ayeh-Kumi PF, Ali IM, Lockhart LA, Gilchrist CA, Petri WA Jr, Haque R. Entamoeba histolytica: genetic diversity of clinical isolates from Bangladesh as demonstrated by polymorphisms in the serine-rich gene. Exp Parasitol. 2001;99(2):80–8. 10.1006/expr.2001.4652 11748961

[27] Haghighi A, Kobayashi S, Takeuchi T, Thammapalerd N, Nozaki T. Geographic diversity among genotypes of Entamoeba histolytica field isolates. J Clin Microbiol. 2003;41(8):3748–56. 10.1128/JCM.41.8.3748-3756.2003 12904386 PMC179867

