# Correction: Prevalence of cases of amebic liver abscess in a tertiary care centre in India: A study on risk factors, associated microflora and strain variation of *Entamoeba histolytica*

**DOI:** 10.1371/journal.pone.0215774

**Published:** 2019-04-17

**Authors:** The PLOS ONE Staff

Due to an error introduced during the typesetting process, Fig 4 is incorrect. The publisher apologizes for the error. Please see the correct Fig 4 here.

**Fig 4:**
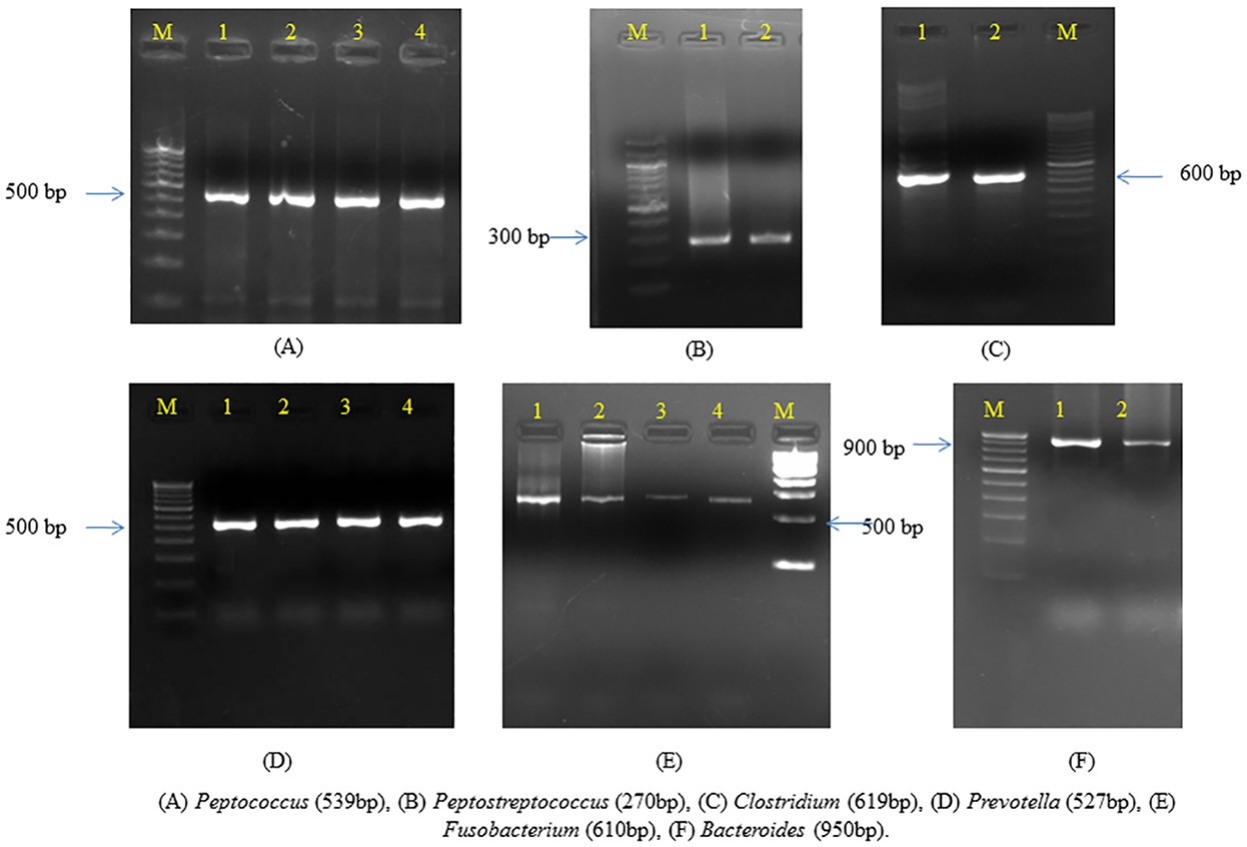
Agarose gel electrophoresis of different anaerobes in liver abscess samples.
