# Supplementary material for: Prevalence of cases of amebic liver abscess in a tertiary care centre in India: A study on risk factors, associated microflora and strain variation of Entamoeba histolytica
Source: PLoS One. 2019 Apr 3;14(4):e0214880. doi: 10.1371/journal.pone.0214880 (PMC6447230; doi:10.1371/journal.pone.0214880)
Supplement: S1 Supporting Information — (PDF) [file pone.0214880.s002.pdf]

Form No.: MB/AS/____

Case report questionnaire

Study on assessment of risk factors in cases of amebic liver abscess

Demographic details

S.No.	Item	Value
1.	Date:	D D / M M / Y Y Y Y
2.	Patient's name:	First name / Middle name / Surname
3.	MRD number:	
4.	Age:	years
5.	Sex:	Male ☐ Female ☐
6.	Phone number:	
7.	Other contact number:	
8.	Address:	Block:<br>Village:<br>District:<br>State:<br>Pin code:

Personal details

9. Education:	Illiterate ☐	Primary ☐	Higher ☐	
10. Occupation:	Skilled ☐		Unskilled ☐	
11. Alcoholism:	YES ☐		NO ☐	
12. Drinking water:	Treated ☐		Untreated ☐	

Clinical characteristics

13. Liver lobe: Right ☐ Left ☐

14. Count of abscess: Single ☐ Multiple ☐

15. Size of abscess: ☐☐☐ cm

16. Pus color: Anchovy ☐ Others ☐

17. Symptoms: Abdominal pain in right upper quadrant ☐ Fever ☐

Vomiting ☐ Weight loss ☐ Diarrhea ☐ Constipation ☐

Any other (Please specify) ☐

Blood profile

18. Alkaline phosphatase: ☐☐☐☐ U/L

19. White blood cells count: ☐☐☐ $\times 10^3/\mu l$

20. Hemoglobin: ☐☐.☐ g/dL

फ़ॉर्म संख्या: एमबी/ एस/_____

केस रिपोर्ट प्रश्नावली

एमीबिक लीवर ऐब्सेस की स्थिति में संभावित खतरे के कारकों के आकलन का अध्ययन

जनांकिकीय विवरण

1. दिनांक
2. मरीज़ का नाम:
3. MRD क्रमांक:
4. उम्र: वर्ष
5. लिंग: पुरुष स्त्री
6. फ़ोन नं.
7. अन्य सम्पर्क नं.
8. पता:

ब्लॉक
गाँव
जिला
राज्य
पिनकोड

व्यक्तिगत विवरण

9. शिक्षा: अशिक्षित प्राथमिक उच्च
10. व्यवसाय: कुशल अकुशल
11. शराब की लत: हाँ नहीं
12. पीने का पानी: उपचारित अनुपचारित

क्लीनिकल/ नैदानिक विशेषताएँ

13. लीवर लोब: दायाँ ☐ बायाँ ☐

14. ऐब्सेस/घाव की संख्या: एक ☐ कई ☐

15. ऐब्सेस/घाव का आकार: ☐☐☐ सेमी

16. मवाद का रंग: ऐन्चोवी ☐ अन्य ☐

17. लक्षण: पेट के दायें ऊपरी भाग में दर्द ☐ बुखार ☐ उलटी ☐

वजन घटना ☐ डायरिया ☐ कब्ज़ ☐

कुछ अन्य (कृपया बताएं) ☐

रक्त का विवरण

18. एल्कलाइन/ क्षारीय फॉस्फेटेज: ☐☐☐☐ यू/ली0

19. सफ़ेद रक्त कणिकाओं की संख्या: ☐☐☐ $\times 10^3$/ माइक्रो-लीटर

20. हीमोग्लोबिन: ☐☐.☐ ग्रा / डीएल
